# Hadza Color Terms Are Sparse, Diverse, and Distributed, and Presage the Universal Color Categories Found in Other World Languages

**DOI:** 10.1177/2041669516681807

**Published:** 2016-12-01

**Authors:** Delwin T. Lindsey, Angela M. Brown, David H. Brainard, Coren L. Apicella

**Affiliations:** Ohio State University, Columbus, Mansfield, OH, USA; Ohio State University, Columbus, OH, USA; University of Pennsylvania, Philadelphia, PA, USA

**Keywords:** color naming, color, Hadza, individual differences, Somali

## Abstract

In our empirical and theoretical study of color naming among the Hadza, a Tanzanian hunter-gatherer group, we show that Hadza color naming is sparse (the color appearance of many stimulus tiles was not named), diverse (there was little consensus in the terms for the color appearance of most tiles), and distributed (the universal color categories of world languages are revealed in nascent form within the Hadza language community, when we analyze the patterns of how individual Hadza deploy color terms). Using our Hadza data set, Witzel shows an association between two measures of color naming performance and the chroma of the stimuli. His prediction of which colored tiles will be named with what level of consensus, while interesting, does not alter the validity of our conclusions.

We recently examined the color terms used by the Hadza, a nomadic hunter-gatherer society in Tanzania (Lindsey, Brown, Brainard, & Apicella, 2015), eliciting color terms using a set of Munsell color tiles. In his commentary on that work, Witzel (2016) argues that the conclusions we draw about the evolution of color terms “could be due to variations in saturation in the stimulus set rather than being evidence for universal constraints on colour term evolution.” Witzel’s argument is based on evidence that the nameabilty in our data set of a tile’s color appearance, and the between-informant consensus in terms used to name a tile, are related to the tile’s chroma (saturation; see also Witzel, Cinotti, & O’Regan, 2015, Figure 5, for a similar analysis). We agree with Witzel that stimulus choice is an important consideration in the study of color naming, but we diverge on the specific point that the way we sampled color space through our choice of Munsell color tiles is problematic for our conclusions.

## Sparse, Diverse, and Distributed

Color naming among the Hadza exhibited three properties. First, color names were sparse: Every informant left the colors of at least some tiles unnamed, and the average informant named only 11.7 (49%; *SD* = 4.2) of the 23 color tiles in the stimulus set. Second, color terms were diverse across speakers: Most of the tile colors received many different color terms, with an average of 8.1 (*SD* = 2.9) color terms per tile, not including “don’t know,” which was the most common response. Third, the universal color categories, which are present in world languages (Kay, Berlin, Maffi, Merrifield, & Cook, 2010; Lindsey & Brown, 2006), are also present, in distributed form, in the Hadzane data set. Of the Hadzane lexical color groups (LCGs) of tile colors receiving the same term by a given informant, 74% fell wholly within single universal color categories (Figure 2(g) of Lindsey et al., 2015). Although no single Hadza informant showed a complete representation of all the universal categories, each informant provided LCGs falling within his or her own, distinct subset of the universal categories. Thus, the categories were distributed across the idiolects of our sample of Hadza informants. Our Somali data set was also sparse, diverse, and distributed (88% of tile colors named, 8.39 terms per tile, and 85% of Somali LCGs within universal categories), suggesting that these are general properties of world languages and not an idiosyncrasy of the Hadzane language. Overall, our results suggest that color term evolution occurs as people communicate with one another, and come to agree on what the color categories are, which colors fall into each category, and what color terms should name them. Our results are important because they elucidate the nature of color naming in a language that is near the beginning of this evolutionary process.

## Stimulus Chroma and “Chroma Contrast”

**Figure 1.**
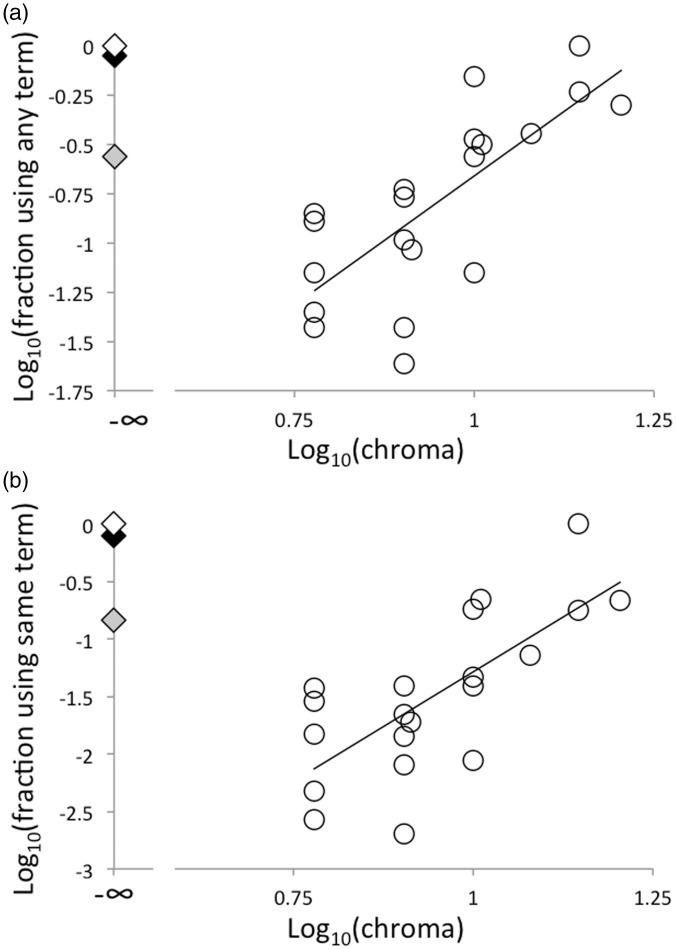
Nameability (a reciprocal index of sparsity) and consensus (a reciprocal index of diversity) as a function of stimulus chroma (an index of saturation) for Hadza informants. A few data points have been jittered in chroma for visibility. Linear regression lines fitted to the chromatic data (circles) are both statistically significant: (a) *r* = .72, *p* = .0004; (b) *r* = .74, *p* = .0002. Diamonds: black, white, and gray stimuli excluded from this analysis.

Witzel claimed that the likelihood that a tile would be named was directly related to its “chroma contrast,” which he defined as the absolute difference between a tile’s chroma and the median chroma of the stimulus set. We have reexamined Witzel’s claim (Figure 1), and we confirm that the nameability and consensus indices are associated with the chromas of the tiles, but we see little reason to choose “chroma contrast” rather than the more standard chroma as the independent variable of interest. Notice, however, that the result shown in Witzel (2016) and in Figure 1 address which tiles will receive color names, and whether informants will agree about the name, but it does not address how these named LCGs will be distributed across the data set.

## Stimulus Choice

The choice of stimuli is a well-known problem in ethnographic research (Rosch Heider, 1972). If the stimuli in an experiment fail to span a wide range of the hues, saturations, and brightnesses of visible stimuli, it is likely that the full range of color naming behavior will not be revealed. Therefore, we sampled the Munsell Book of Color to include both tiles whose colors are highly saturated (e.g., red, orange, magenta, and cyan) and not highly saturated (e.g., olive, brown, and lavender), and tiles whose perceived colors are named both with high consensus (e.g., red, yellow, and green) and with low consensus (e.g., peach, mustard, and cyan) in world languages. Inasmuch as the Munsell stimulus set used in most studies of color naming contains chromatic colors that fall on a two-dimensional manifold within three-dimensional color space, there were necessarily colors that were not represented. For example, there was no chromatic stimulus at middle brightness but low saturation.

In his critique of our study, Witzel argues that our results were somehow foreordained by our color sampling strategy and would have been substantially different had we chosen a different stimulus set. Had we sampled color at only low or only high saturation, we would probably have obtained different quantitative results: We might have obtained consistently lower or consistently higher values of sparsity and diversity, but we would probably have missed the range of our informants’ color naming behavior. In a similar spirit, had we sampled colors only at a fixed hue, but different lightnesses and chromas, that would certainly have altered our results: We would probably have missed most of the color naming diversity across the Hadza language community, as well as among the speakers of Somali and English. Note, therefore, that the key factor driving our conclusions about diversity and sparsity is the comparison of color naming across Hadzane, Somali, and English for a common stimulus set.

Overall, we thought it wise, particularly given the limited time we had available with each Hadza informant, to follow the advice of Rosch Heider, and sample a wide range of perceived hues, saturations, and lightnesses, recognizing that it was not feasible to employ a fully crossed (Hue × Saturation × Lightness) experimental design. We chose a subset of the color tiles from the World Color Survey (Kay et al., 2010) so that that we could compare our results to that large extant data set. We do not disagree with the general possibility that future studies of color naming, with larger or different stimulus sets, could increase our understanding of color naming, color categories, and color term evolution: The possibility that different results would occur under different conditions from those used in any experiment is always present in science. However, we diverge from Witzel in that we do not believe our stimulus set was unfortunately chosen in any obvious way that would bias the data in support of our claims, given current knowledge taken together with the analysis shown by Witzel and in Figure 1: Hadza color naming is sparser and more diverse than Somali color naming, which in turn is sparser and more diverse than English color naming. This would likely remain true even if higher nameability and consensus were to be observed within the Hadza for stimuli with higher chroma.

## Distributed Representation of Color Categories

The third key result of our study, the lawful distribution of color terms across our stimulus set, depends on how the perceived colors of our tiles were actually named, not merely on whether they were named, or with what consensus they were named. Our Hadza informants grouped the colors of our tiles into LCGs that respected the boundaries of the universal color categories revealed in previous research (Lindsey & Brown, 2006). This can be seen in Figure 2(a) Lindsey et al. (2015), where few of the LCGs (coded in gray) spanned more than one universal category. Like the universal color categories, the Hadza LCGs generally covered narrow hue ranges (in our Figure 2(a) and (d)) but often a wide range of chromas. Only 6% of Hadza LCGs contained tiles of the same Munsell chroma, while 58% included tiles spanning four or more chroma units. The narrow hue range of each LCG is why the autocorrelation of our data is highest near zero rotation parallel to the hue dimension (in our Figure 2(g)). The results in Witzel and in Figure 1 do not speak to these properties of the data, which are at the heart of our third result: Color categories are represented in a distributed manner within our data set. It is not material to this conclusion or its implications for color term evolution whether color naming occurs with higher consensus for colors that we did not study.

## Conclusions

We applaud Dr. Witzel for providing a falsifiable hypothesis that might predict which particular color tiles will be named with what particular levels of frequency and consensus. These are interesting questions, but not ones that we spoke to in our article. Furthermore, even if the hypothesis turned out to be correct, that would not alter any of our conclusions: The perceived colors of our tiles are named sparsely across the Hadza and Somali data sets, with low overall consensus, and the universal color categories found in the World Color Survey are distributed in the idiolects within the Hadzane and Somali language communities rather than being clearly represented in the responses individual informants.
